# Understanding the burden of congenital cytomegalovirus (cCMV) infection: concept elicitation interviews with caregivers of pediatric cCMV patients and development of a conceptual disease model

**DOI:** 10.1007/s11136-025-04142-3

**Published:** 2026-03-01

**Authors:** Brooke M. Currie, Georges Van Kriekinge, Aurélie Pimienta, Cory D. Saucier, K. D. Jacobs, Laura Tesler Waldman, Christine M. Salvatore

**Affiliations:** 1 GSK, Collegeville, PA USA; 2 GSK, Wavre, Belgium; 3 QualityMetric, Johnston, RI USA; 4 Weill Cornell Medicine, New York, NY USA; 5 Present Address: RTI Health Solutions, Ann Arbor, MI USA; 6 Present Address: Lumanity Patient-Centered Outcomes LLC, Boston, MA USA

**Keywords:** Caregiver, Congenital cytomegalovirus, Quality of life, Interviews, Impact

## Abstract

**Purpose:**

Congenital cytomegalovirus (cCMV) is the most common congenital infection worldwide. cCMV can result in long-term impairments such as hearing loss and developmental delay. The study objective was to understand the caregiver experience of cCMV and caregiver-reported perceptions of the experience for cCMV patients.

**Methods:**

This non-interventional, cross-sectional study interviewed 25 caregivers of patients (aged 4 months to 17 years) with confirmed cCMV in the United States. Semi-structured qualitative interviewing methods were used to elicit descriptions of cCMV symptoms and their impact on the patient as perceived by the caregiver. Separate questions assessed the caregiver burden of a patient with cCMV. Transcripts were analyzed using an iterative coding process to identify key concepts.

**Results:**

All caregivers reported increased healthcare needs for their child with cCMV. Other caregiver-reported patient impacts spanned multiple domains including emotional, physical and social functioning, education and communication. For example, a child with cCMV-related communication/hearing difficulties experiencing sadness or social isolation, or with cCMV-related seizures resulting in worry or apprehension. The caregiver impacts of caring for a child with cCMV also covered multiple domains including emotional, role and social functioning, work, sleep, and family planning. Emotional functioning was considered the greatest impact by approximately half of caregivers.

**Conclusion:**

These results advance understanding of experiences related to cCMV for caregivers and caregiver-reported perceptions of the patient experience. This may help healthcare professionals improve routine clinical practice for families affected by cCMV. The results and conceptual model may also support the development of a disease-specific clinical outcome assessment tool.

**Supplementary Information:**

The online version contains supplementary material available at 10.1007/s11136-025-04142-3.

## Abstract (plain language summary)

Cytomegalovirus is a common virus, usually mild in healthy adults, that can be passed to an unborn baby during pregnancy (congenital cytomegalovirus). Some babies born with congenital cytomegalovirus have long-term health problems, such as hearing loss or learning disabilities. This study interviewed 25 parents caring for children with congenital cytomegalovirus in the United States to explore the impact of the disease on the lives of patients and caregivers.

Impacts of congenital cytomegalovirus on the children, as reported by the caregivers, included emotional (e.g., anxiety, fear, worry), physical (e.g., needing devices to assist hearing) or social effects (e.g., social withdrawal), or difficulties with education or communication.

All caregivers reported emotional impacts such as feelings of shock, anxiety, and fear, sometimes lasting a long time as parents worried about potential future impacts of congenital cytomegalovirus on their child. These results help to understand the impacts of congenital cytomegalovirus on the lives of patients and their families.

**Supplementary Information:**

The online version contains supplementary material available at 10.1007/s11136-025-04142-3.

## Introduction

Cytomegalovirus (CMV) is a human-specific herpes virus [1]. It is common in people of all ages and infection is lifelong [2]. Over half of adults in the United States (US) are estimated to have been infected with CMV by the age of 40 years, and most infections are asymptomatic [2]. CMV can be transmitted from mother to fetus during pregnancy [3], and congenital cytomegalovirus (cCMV) is the most common congenital infection worldwide [1]. The global prevalence of cCMV has been estimated at 0.67%, with higher prevalence rates in low- and middle-income countries (1.42%) than high-income countries (0.48%) [4]. In the US, it is estimated that approximately 1 in 200 infants is born with cCMV [3], and that almost one in three children has been infected by the age of five years [2].

At birth, 85–90% of infants with cCMV are asymptomatic [1]. For the remaining 10–15% of cases, signs at birth may include rash, jaundice, hepatosplenomegaly, and microencephaly [3]. Approximately half (40–58%) of infants born with visible signs and 13.5% without signs/symptoms at birth are estimated to develop long-term sequelae, including hearing loss, developmental delay, learning disabilities, and behavioral disorders [5–9]. cCMV is the leading non-genetic cause of sensorineural hearing loss in children, and the leading viral cause of neurodevelopmental delay [1]. A study of 26 children with cCMV infection in Sweden reported that cCMV affected general brain development, resulting in a complex pattern of difficulties, including disturbances in balance, delayed walking, and feeding difficulties for some children [8].

There is no international consensus on screening and/or treatment of cCMV. Guidelines have been published in Canada [10], Europe, and Japan [11; 12], and some recommendations have been made by an informal international group [13]. The province of Ontario, Canada, has added CMV to its universal newborn screening tests, as an adjunct to the established newborn hearing screening program [10]. In the US, clinical practice patterns and guidelines vary [14]. Some states have mandated early targeted cCMV screening based on failed hearing evaluation at birth, and some individual hospitals have implemented screening programs [15]. Clinical evidence supporting the effectiveness of cCMV treatments remains limited, and there is a lack of consensus regarding appropriate cCMV treatment approaches among healthcare providers [16].

Studies on the humanistic impact of cCMV on patients and their caregivers are currently limited. Three US healthcare professionals who are also mothers of children with cCMV have described challenges, including managing numerous medical appointments and home therapy regimens, loss of work productivity and career ambitions, and effects on relationships with other family members [17; 18]. Emotional impacts reported by these authors included feelings of fear about the future, as well as guilt or self-blame due to the theoretically preventable nature of cCMV [18]. One mother of a baby with cCMV described her feelings in the early months following her child’s diagnosis using intensely emotional terms, ‘I was exhausted and overwhelmed’, and ‘…when I looked at my baby, in place of love, I felt regret, worry, guilt, and bitterness’ [19].

Health-related quality of life (HRQoL) is a general concept in health outcomes research that refers to physical, mental and social aspects of functioning and well-being [20]. HRQoL is usually self-reported by patients [20], but can be reported by proxy respondents such as parents if patients cannot self-report, for example in young children or patients with cognitive impairment [21].

The objective of the present study was to characterize the experience of cCMV and its short- and long-term sequelae for caregivers and for patients as reported by caregivers, using qualitative interviewing methods.

## Methods

This non-interventional, cross-sectional concept elicitation study used semi-structured qualitative interviewing methods to characterize concepts related to the caregiver-assessed patient and caregiver experience of cCMV, including impacts on HRQoL.

A previously conducted unpublished systematic literature review (SLR) was used to inform the development of an initial conceptual disease model to illustrate all important concepts related to patients’ and caregivers’ experience of cCMV and map the relationships between the concepts. Concepts included disease signs and symptoms, impacts on activities of daily living, and HRQoL. The conceptual model also included modifying factors, items that can worsen (negative) or mitigate (positive) HRQoL impacts. This unpublished SLR found no disease-specific clinical outcome assessment tools for cCMV, and identified one qualitative study evaluating impacts of cCMV on patients and caregivers [22].

Later, a further follow-on targeted literature review (TLR) was conducted to update and expand the conceptual model derived from the SLR to develop a preliminary conceptual model (Figure S1). To conduct this TLR, PubMed was searched on 26 May 2022 for articles reporting concepts related to the burden of disease for patients with cCMV and their caregivers, and records were screened using a two-step process with prespecified inclusion criteria (Table S1). As well as informing revisions to the conceptual model developed from the earlier SLR, the TLR results were also used to guide the development of the interview guide and other study materials for the qualitative research described herein.

An interview guide was developed based on the TLR results and clinical expert input, with open-ended questions to elicit descriptions of cCMV symptoms and their impact on patients’ lives as perceived by the caregivers. A separate set of interview questions in the guide assessed the burden of caring for a patient with cCMV. The project team consisted of health outcomes researchers with academic backgrounds in public health, psychology, and anthropology (Supplementary Information S1).

Interviews were conducted by two trained interviewers (authors KDJ and CDS) between 10 January 2023 and 17 February 2023. Interviewers used their discretion to probe further into participants’ responses to obtain clarification and further information. Interviews lasted 60–75 min and were conducted using Health Insurance Portability and Accountability Act (HIPAA)-compliant Zoom videoconferencing software, with the option of webcam or telephone use depending on the participant’s technical capabilities and comfort level. For more information, see Supplementary Information S1.

All interviews were audio-recorded following appropriate caregiver informed consent and transcribed verbatim.

The study was conducted in the US with 25 caregivers of children with confirmed cCMV. Participants were recruited by an organization specialized in recruitment services, using purposive sampling to identify and enroll caregivers of cCMV patients in six age ranges: infants (aged 0–11 months); toddlers (aged 1–3 years); pre-school children (aged 4–5 years); pre-adolescents (aged 6–11 years); adolescents (aged 12–17 years); adults (aged 18 + years). Potential participants were identified from a pre-existing panel, recruiter databases and physician referrals, and were screened by telephone using a study-specific screener to confirm that they met the eligibility criteria. Caregivers were eligible if they were: aged 18 + years; primary caregiver and legal guardian of a patient with cCMV; currently living in the US; able to speak and write English fluently; able to provide confirmation of the patient’s cCMV diagnosis. A primary caregiver was defined as a parent of a patient with cCMV and who had lived with the patient since birth. The patient being cared for must have experienced at least one sign, symptom, and/or long-term complication of cCMV. Two caregivers of the same patient were not permitted to participate to avoid redundant information. No subjects refused to participate or dropped out.

Quality control was conducted on the first 10% of transcripts by a researcher reading the transcript while listening to the interview. Transcripts were coded and analyzed using an iterative coding process entailing content thematic analysis and grounded theory analysis methods [23; 24], using NVivo qualitative software (QSR International Pty Ltd, 2018, v. 12.0). An initial coding structure was developed based on the interview guide concepts identified from the TLR. Subsequently, the coding structure was iteratively refined based on emergent themes identified in the transcripts, permitting a more nuanced understanding of existing concepts, and the incorporation of new ones. The study team analyzed both the frequency of concepts elicited by participants and their reported importance. Information on the coding tree and other study materials is available on request. Two researchers independently coded the first two interviews and compared their coding to assess reliability. Discrepancies were resolved by consensus among the wider study team. Once consensus was reached, the remaining transcripts were coded independently.

Saturation analysis was conducted by grouping transcripts chronologically into five sets of five transcripts each and tracking the number of new codes or concepts emerging in each set. By the third set of transcripts (15 interviews), 97% of concepts had been identified, indicating that 25 participants was a sufficient sample size.

Following analysis of interview results, the preliminary conceptual model was refined by adding additional concepts described by caregivers in relation to signs and symptoms of cCMV infection, patient-experienced short- and long-term complications (as reported by caregivers), patient-experienced impacts on HRQoL as reported by caregivers, caregiver-experienced impacts on HRQoL, and potential modifying factors. Further, general terms within the preliminary model were replaced with specific concepts elicited from caregiver interviews (e.g., “emotional problems” was further refined using more precise terms such as “sadness”). Lastly, concepts that were not supported by either the literature or the caregiver interviews were reviewed to determine their appropriateness within the final model; concepts found to be redundant or unsupported by the literature review or the caregiver interviews were removed, and areas of the conceptual model were regrouped to improve cohesiveness.

*Patient and public involvement*.

Neither the public nor patients were involved in the design of the study.

## Results

### Targeted literature review

The TLR yielded 11 articles that met the inclusion criteria (Figure S2). Patient- and caregiver-experienced impacts on HRQoL identified in the TLR were tabulated, together with potential modifying factors (Table S2).

### Sample characteristics

Table 1 summarizes the demographic characteristics of the sample of interviewees. All participating caregivers were a parent of the patient with cCMV for whom they provided care. Although eligible, no caregivers of adult cCMV patients (aged 18 + years) were recruited into the study. Most caregivers (*n* = 21, 84%) reported that their child was tested and/or diagnosed with cCMV in the hospital shortly after birth. For those whose child was not tested in the hospital shortly after delivery (*n* = 4, 16%), diagnoses were most often made within a few weeks after birth, most commonly at the first newborn follow-up appointment.

**Table 1:** Demographic characteristics of the caregivers and the patients with cCMV

Demographics	Number (*n* = 25)	
	Caregiver demographics, *n* (%)	Caregiver-reported patient demographics, *n* (%)
*Gender*		
Female	14 (56.0)	12 (48.0)
Male	8 (32.0)	10 (40.0)
Do not wish to answer	3 (12.0)	3 (12.0)
*Race/ethnicity* ^a^		
White or Caucasian	11 (44.0)	11 (44.0)
Black or African American	3 (12.0)	3 (12.0)
Hawaiian or Other Pacific Islander	2 (8.0)	2 (8.0)
Hispanic or Latino	2 (8.0)	2 (8.0)
American Indian or Alaska Native	1 (4.0)	1 (4.0)
Do not wish to answer	7 (28.0)	7 (28.0)
*Employment* ^b^		
Full-time	9 (36.0)	
Part-time	1 (4.0)	
Student	1 (4.0)	
Unemployed	15 (60.0)	
*Education*		
Bachelor’s degree	12 (48.0)	
Associate degree	6 (24.0)	
Some undergraduate	3 (12.0)	
High school or GED	2 (8.0)	
Do not wish to answer	2 (8.0)	
*US geographical region*		
West	14 (56.0)	
South	6 (24.0)	
North-east	3 (12.0)	
Mid-west	2 (8.0)	
	Mean (standard deviation)	Mean (standard deviation)
Age, years	41.6 (9.8)	7.1 (5.8)
	Range	Range
	25–56 years	4 months–17 years

^a^ One caregiver reported White or Caucasian and Hispanic or Latino; one caregiver reported their child as White or Caucasian and Hispanic or Latino

^b^ One caregiver worked part time and was a student

cCMV, congenital cytomegalovirus; GED, General Educational Development; US, United States

### Signs and symptoms reported

Table 2 summarizes the signs and symptoms of cCMV spontaneously reported by caregivers at or after birth. Some signs/symptoms, such as low birth weight/small for gestational age, rash, and jaundice were reported as present at birth, whereas others such as vision impairment, hearing loss and cognitive/learning or motor delays were reported as issues that emerged over time, sometimes many years later. For example, one parent reported that the child’s vision impairment appeared at the age of nine years.

**Table 2:** Signs and symptoms of cCMV reported by caregivers

Sign or symptom	Number (*n* = 25)	%
Low birth weight/small for gestational age	10	40.0
Rash	9	36.0
Motor delay	8	32.0
Vision impairment	8	32.0
Cognitive and/or learning delay	5	20.0
Hearing loss	5	20.0
Jaundice	5	20.0
Abnormal liver function	4	16.0
Microencephaly	4	16.0
Language delay	3	12.0
Seizures	3	12.0
Splenomegaly	3	12.0
Perception of a weakened immune system	2	8.0
Abnormal kidney function	1	4.0

cCMV, congenital cytomegalovirus

### Health-related quality of life impact on patients as reported by caregivers

Table 3 summarizes the impacts of cCMV on patients’ lives as reported by caregivers, with example quotations to illustrate the caregivers’ perceptions of the patient experience. All caregivers reported increased healthcare needs for their child with cCMV, including regular monitoring to track disease progression over time, and some children required physical, occupational and/or speech therapy. The caregivers reported that their children with cCMV experienced extensive impacts on their physical functioning (*n* = 11, 44%; e.g., need for an assistive device), social functioning (*n* = 11; 44%; e.g., participating in social activities), emotional well-being (*n* = 10, 40%; e.g., feelings of anxiety, fear, or worry), education (*n* = 9, 36%), and communication (*n* = 8, 32%).

**Table 3:** Health-related quality of life impacts of cCMV on patients, as reported by caregivers

Impact	Number (%) *n* = 25	Illustrative quotations
Healthcare resource utilization	25 (100)	*Uh*,* just*,* you know*,* just normal standard testing. Uh*,* just keeping*,* basically being monitored*,* uh*,* just to keep update date on his condition in case anything unusual comes or any other symptoms come*,* taking blood tests and*,* uh*,* you know*,* vision tests and things like that… Uh*,* I’d say pretty regular*,* regularly*,* like every few weeks…* *Oh*,* in the beginning? I mean*,* I felt like we were there twice a week. I mean*,* they were – you know*,* we’re in the hospital for a while so that took a lot of the back and forth or you know. Just seeing the baby*,* uhm*,* it’s hard. But – so*,* that taken care of the hospital*,* then I*,* I would say one to two times a week probably for the first three months after that just for check-ups… after that part*,* I would say we’ve gone monthly for six months to the six-month mark*,
Physical Functioning Use of assistive device(s) Motor impairment	11 (44.0) 10 (40.0) 3 (12.0)	*We did have to do a cochlear implant*,* uhm*,* almost at five. Because I wanted him to be able to*,* uhm*,* not have any issues in school…* *… so like when he plays with a dog*,* maybe he’s a little bit slower or maybe when he’s a reaction when you tap a shoulder he might takes a little time to react.*
Social Functioning Limiting disclosure of diagnosis Participating in social activities Social Withdrawal Relationships (negative) Relationships (positive)	11 (44.0) 5 (20.0) 5 (20.0) 5 (20.0) 3 (12.0) 2 (8.0)	*She doesn’t mention it… doesn’t want*,* uhm*,* anyone to know as far as outside the family that*,* uhm*,* she has this… It’s nothing that she talks about… I haven’t asked her why hasn’t she talked to her friends about it…* *…we noticed her not responding*,* uhm*,* and not participating*,* uhm*,* in things. Like if the other girls…would say something*,* and she wouldn’t respond*,* and she…wasn’t playing normally…* *And I might be wrong comparing but he’s very reserved*,* you know. Very shy*,* kind of like want to do his own thing and don’t want to*,* sometimes*,* share anything with*,* with anybody else… He’s not the outgoing kid that will just go around and play with everybody else*,* you know. It’s like*,* you have to tell him*,* you know*,* “It’s okay honey*,*” you know. “Go play*,*” you know…* *Uh*,* if [her classmates] don’t understand her*,* they sometimes yell at her. And when*,* uh*,* people act like that*,* I guess that upsets her. And so*,* yes*,* in some ways they*,* they ridicule her of the issues that she’s*,* uh*,* experiencing…* *I feel like*,* uh*,* the teacher saw she needed more*,* uh*,* special*,* uh*,* attention*,* and so they became closer and they actually wanted to help her*,* uh*,* even more…they wanted her to feel*,* uh*,* same as*,* uh*,* other students not be impacted*,* not feel*,* uh*,* depressed about it and*,* uh*,* not be traumatized as much…*
Emotional Functioning Anxiety/fear/worry Resilience (positive) Sadness	10 (40.0) 6 (24.0) 5 (20.0) 4 (16.0)	*…the times that she has had*,* uhm*,* seizures…I think that is more alarming to her*,* you know*,* and frightening to her because*,* uhm*,* it has such a sudden*,* uhm*,* onset…we’ve talked about it*,* and we – and I think from an emotional standpoint*,* there is a bit of*,* uh*,* worry and a bit of apprehension…* *I know she was sad about the implant… She just says*,* “I feel bad that I have to have this.” And she was very sad. It was sad. But she was able to hear. That was the main thing and I even tell her that. “The main thing is*,* you can hear now.”* *But*,* you know*,* she realized that she’s got difficulties*,* uh*,* uh*,* uh*,* for herself*,* but then she also asks*,* uh*,* positively that she can overcome any barrier that is placed upon her*,* and she’s*,* she works hard to overcome her challenges…*
Education Accommodations Modifications	9 (36.0) 9 (36.0) 2 (8.0)	*Uhm*,* but the thing is*,* when she lost her hearing and she was going through the processes of losing it*,* she read more. She read a lot. She’s a big reader. And*,* uhm*,* I think that helped her not to fall behind in studies because she made good grades…I think she learned more once she got home*,* uhm*,* by going over her studies without even – she*,* she couldn’t hear everything the teacher was saying…* *They test you and*,* um*,* they don’t give you work to overwhelm you…We have to do it every year*,* reassessment every year and just give you different goals that he can implement*
Communication Social skills Expressive and receptive language	8 (32.0) 6 (24.0) 5 (20.0)	*…that upsets her when people*,* uh*,* speak loudly to her or stare at her because she doesn’t really understand*,* and that that’s pretty much the only time that she’s*,* uh*,* upset about*,* uh*,* having*,* uh*,* difficulties communicating…* *I think sometimes he*,* especially with conversational misunderstanding with*,* I guess with me*,* um*,* he will get*,* um*,* defensive*,* um*,* and he would say stuff like*,* oh*,* I understand*,* I understand. You think I don’t understand. I understand. So he’s really defensive at first*,* you know*,* with it all. But then deep down inside*,* I*,* you know*,* I think he doesn’t really understand*,* so.*

cCMV, congenital cytomegalovirus

### Health-related quality of life impact on caregivers

Table 4 summarizes the impacts of their child’s cCMV on caregivers’ lives, with example quotations to illustrate the caregivers’ lived experiences. Caregivers reported a wide range of impacts of caring for a child with cCMV in multiple domains including emotional, role and social functioning, work, sleep, and family planning. All caregivers reported effects on emotional functioning (e.g., anxiety, fear, sadness, stress) and approximately half (*n* = 12, 48%) considered the emotional impact as the greatest effect on their lives of caregiving for a child with cCMV. While sadness and depression lessened over time for many caregivers, 17 (68%) reported experiencing stress that persisted long after the time of diagnosis. Almost all caregivers (*n* = 22, 88%) reported feeling uncertain about the future and worrying about how cCMV would affect the child’s life. Ten caregivers (40%) were employed at the time of the interview, 70% (*n* = 7) of whom reported that caring for a child with cCMV had affected their work. All caregivers reported that the child with cCMV was either the only child or the youngest child in the family. Seven (47%) of the 15 caregivers who were explicitly asked and felt comfortable answering said the risk of having another child with cCMV had contributed to their decision not to have more children.

**Table 4:** Health-related quality of life impacts of cCMV on caregivers

Impact	Number (%) *n* = 25	Illustrative quotations
Emotional Functioning Anxiety/Fear/Worry Uncertainty about the future Stress Sadness Shock at diagnosis Anger/Frustration Guilt/Regret	25 (100) 25 (100) 22 (88.0) 17 (68.0) 15 (60.0) 13 (52.0) 12 (48.0) 11 (44.0)	*But I think at night*,* you know*,* when you’re just resting and your mind is zooming around*,* uhm*,* it goes into places that that stress eats at me*,* uhm*,* and makes*,* you know*,* restless and a little bit*,* you know*,* maybe anxious. I’m concerned about the future and you know*,* I’m just unsettled…* *Yes. I definitely have stress*,* anxiety. I – years ago*,* it was with me daily…* *Oh*,* I was shock in that time*,* uh*,* was almost 14*,* 15 years ago. Uh*,* I was shock in that time. I was not*,* uh*,* expecting this. And*,* uh*,* I was so sad and*,* uh*,* uh*,* and depressed at the first couple of months…* *I was definitely all eyes and ears and absolutely watching my child like a hawk. Like I wasn’t sleeping*,* hence the extreme depression. Um*,* yeah*,* I didn’t even know*,* I mean*,* miserable.* *Like*,* how did this happen? I didn’t know that I had this like*,* more mad*,* more mad at like the medical system*,* and blood testing*,* and*,* you know. We should test everybody for everything*,* right?* *Uhm*,* and then*,* you know*,* sometimes*,* she’ll jump*,* and she’ll go*,* “You know you gave this to me.” You know*,* that type of thing. And we do laugh it off*,* but I do feel kind of hurt behind it. You know? It*,* it’s something that*,* uhm*,* a parent*,* uh*,* feels guilty about. I did feel guilty…*
Social Functioning Relationships (negative) Limiting disclosure of diagnosis Relationships (positive) Participating in social activities	20 (80.0) 12 (48.0) 11 (44.0) 5 (20.0) 2 (8.0)	*…it’s not good for relationships. You know*,* me and my wife can do a lot of arguments and*,* you know*,* almost got divorced. And uh*,* it’s been*,* it’s been tough.* *Uhm*,* well*,* we don’t hang out anymore*,* because*,* uhm*,* always busy with the baby and*,* uhm*,* also I worry that*,* you know*,* taking the baby out*,* she might get sick. So*,* we don’t really take her out to be around a lot of people…* *My husband didn’t tell anybody at his job. Um*,* you know*,* I talk like*,* I think I also told a probably*,* a couple of close friends pretty immediately*,* just like girlfriends of mine. But I didn’t like publish it and broadcast it on like social media…* *Um*,* our relationship is great. It’s stronger. It’s*,* it’s um*,* yeah. I wouldn’t say the stress has affected our relationship at all.* *You focus more on health rather than just having fun. It takes away fun time*,* but at the same time*,* you*,* you just have to make those adjustments… you don’t go on vacations*,* you know…*
Role Functioning Protective/Restrictive Adjustments to schedule/Routine Advocacy	17 (68.0) 9 (36.0) 8 (32.0) 4 (16.0)	*…the immunocompromised always feeling vulnerable…Um*,* but his daily life*,* I mean*,* yeah*,* like today he’s fine*,* but I mean ongoing colds and stuff. So it impacts him like more of if there’s anyone sick*,* I don’t want to go around them*,* um*,* you know…* *Well*,* as far as*,* it*,* it’s a great impact because I had to change my life completely from my first son. Um*,* I’m now at home because*,* um*,* I don’t trust anyone… I’m there to pick him up*,* um*,* drop him off*,* you know*,* stuff like that…* *But*,* you know*,* when you are dealing with*,* uhm*,* a chronic disease like this*,* a chronic illness that this is going to be a lifetime thing. There is no cure for this. Uhm*,* that’s something that my husband and I have had to*,* you know. We want to be prepared. I think we want to be realistic*,* and*,* and how could we*,* you know*,* uhm*,* best serve her and make sure that she’s getting*,* you know*,* the care that she needs that she’ll probably need throughout her lifetime… I mean*,* I’ll always go with her as long as she wants me to. I will always be her advocate…I am her caretaker*
Financial Medical costs	13 (52.0)	*Oh*,* it’s been expensive… We have doctor spending money*,* taking time. I have sometimes ditch work and*,* and it’s just*,* it’s a lot of*,* it’s financially stressful. Sure. I mean*,* yeah*,* be honest of course. Because you like a juggling act…* *…hiring a therapist for speech therapy. Because for some reason*,* the insurance don’t cover it*,* you know. For some reason*,* because I guess it’s something to do with specialty services*,* so it doesn’t cover speech therapy.*
Sleep Interference with sleep	8 (32.0)	*I’ll be like*,* “Gosh*,* why am I so tired?” You know? And it will happen on occasion. Uhm*,* you know*,* and*,* like I said*,* if I’m not – if I’m kind of burning the candle at both ends*,* I’m not getting enough sleep. Uhm*,* you know*,* I’m kind of overextending myself. Uhm*,* that’s when I notice it*,* you know. I’ll be like*,* “Gosh*,* why am I so tired?”*
Work Modifications Productivity Absenteeism	8 (32.0) 5 (20.0) 3 (12.0) 1 (4.0)	*I had to…make adjustments and work from home instead of going out to an office. It has impacted a little bit of my social life being a caretaker… My – pretty much my – almost my entire life kind of evolves around my child now…*
Family Planning Decision not to have additional children	7 (28.0)	*I think the only other thing I probably mentioned was that my husband and I have officially shut the door in having more children after this… [cCMV] had something to do with it. I mean*,* we were on the fence about having another. But I think after this experience of knowing that*,* again*,* how the…you know*,* the baby got it from me*,* um*,* we just don’t want to take any other additional risks*
Physical Functioning Exhaustion/Fatigue	4 (16.0)	*Total exhaustion when I’m – because I*,* I don’t wake up as energetic when – even I have a good night’s sleep. So*,* yeah*,* a little bit of exhaustion. Yep [it is related to cCMV]*

cCMV, congenital cytomegalovirus

### Modifying factors

The interviews indicated seven potential factors that moderated the degree of impact for the patient with cCMV and/or their caregiver, where the degree of impact was either lessened or exacerbated depending on how the factor was experienced (Table S3). For example, medical professionals’ knowledge and awareness of cCMV, particularly at time of diagnosis, was mentioned as a positive experience by nine caregivers (36%) and a negative experience by eight (32%), and medical support as a positive experience by four (16%) and a negative experience by two caregivers (8%). None of the parents had prior knowledge of cCMV, and 13 (52%) reported feelings of shock when they received their child’s diagnosis. Time since diagnosis, another potential modifying factor, was raised by 18 (72%) of caregivers. Most (*n* = 17, 68%) reported that the initial shock or worry diminished with time since diagnosis. However, six (24%) reported concerns worsening over time after the initial shock, such as worry over disease progression.

Approximately half the sample (*n* = 13, 52%) found health insurance to be sufficient for the care their child was receiving, although one (4%) stated that insurance did not adequately cover necessary services such as speech therapy. Thirteen (52%) reported increased medical costs with cCMV, and four (16%) stated that the increased medical costs were burdensome.

### Final conceptual model

**Fig. 1:**
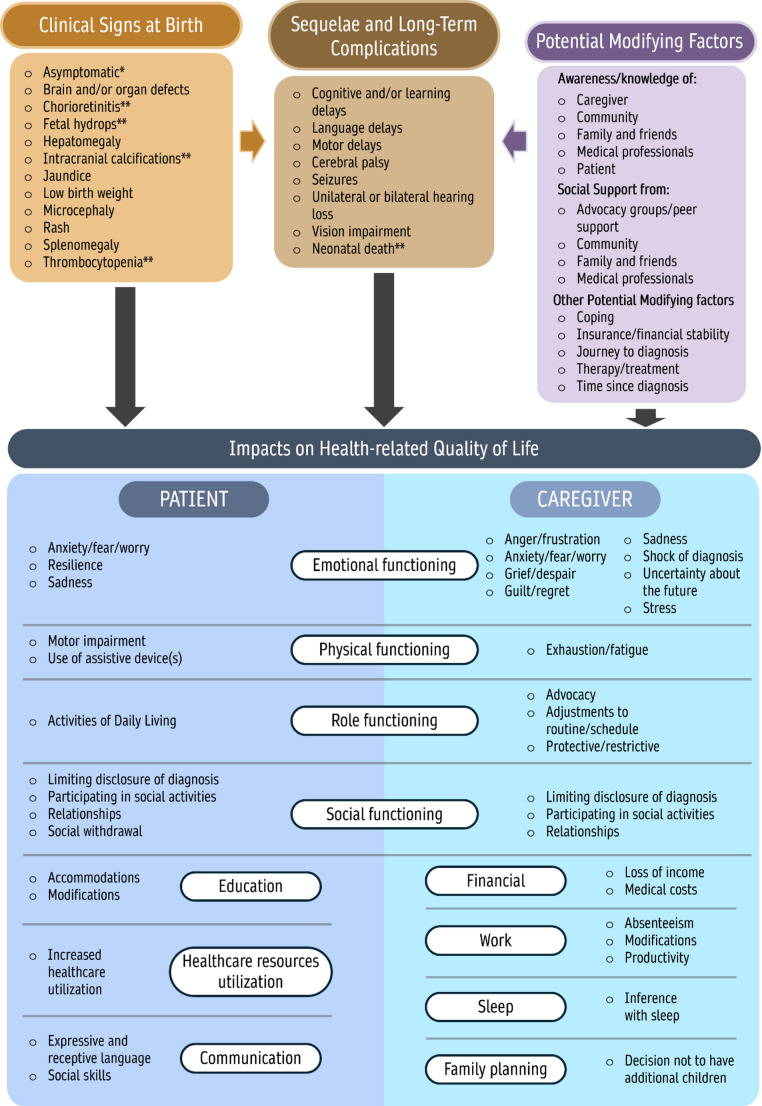
Final conceptual model - Impacts of congenital cytomegalovirus (cCMV) on patients as reported by caregivers and on caregivers. This conceptual model illustrates the clinical signs at birth, sequelae, and long-term complications associated with cCMV. It highlights the multifaceted impacts on health-related quality of life (HRQoL) for patients as reported by caregivers and for caregivers, including emotional, physical, role, and social functioning. Additional domains, such as education, healthcare resource utilization, financial costs, sleep, work, and family planning, are shown to further affect patients, as reported by caregivers, and caregivers. Modifying factors, such as awareness, social support, and coping mechanisms, influence the overall experience of managing cCMV. Impacts on HRQoL include impacts reported by caregivers for themselves and by caregivers for patients, and refers to impacts reported at any stage of life (not limited to the newborn stage). Data are based on caregiver interviews and literature review. * Fowler et al. (2018) [25]. ** indicates findings present in the original model or elicited by a key medical expert but not reported in the TLR literature review or caregiver interviews

The final conceptual model is shown in Fig. 1. Concepts were selected for inclusion based on the degree of support in the literature and the proportion of caregivers who reported the concept in the interviews. The model includes six HRQoL domains related to the impacts on patients as reported by caregivers plus increased healthcare resource utilization, eight HRQoL domains related to the impacts on caregivers, and seven modifying factors (awareness/knowledge of cCMV, social support, and five other factors).

## Discussion

The study findings indicate that cCMV is associated with a wide range of clinical signs/symptoms. Some, such as low birth weight or jaundice, may resolve over time, while others emerge later. Caregivers reported multidimensional impacts on HRQoL, including emotional, social and physical functioning, for patients, and for caregivers themselves. All patients required increased healthcare needs, such as regular monitoring visits, treatments and/or therapy, or assistive devices. The wide range of experiences reported in this study is an important contribution to understanding the burden of cCMV.

Almost half (*n* = 7, 47%) of the 15 caregivers who were asked and felt comfortable answering said the risk of having another child with cCMV had contributed to their decision not to have more children. Pre-specified probes related to family planning were added to the discussion guide after nine of the interviews had been conducted, once it became clear that this was a consistent theme emerging from the earliest interviews. Thus, this topic was not systematically covered across all participants and the result reported here may be an underestimate of this important impact.

The results of this study are broadly consistent with previously published research. In a US study, parents reported feelings of shock and dismay at the initial cCMV diagnosis [26]. Parents also wanted to know the likely short- and long-term health outcomes for their child, and desired information on signs and symptoms to watch out for to help reduce unnecessary delays in development [26]. Another US study reported that parents experienced distress and anxiety at the initial diagnosis, especially related to uncertainty about the long-term prognosis, but felt it was important to know so that they would be better able to make informed decisions [27]. Both hearing loss and vestibular insufficiency (which can manifest as delays in motor development, emotional and social development, and cognition) can be late-onset, progressive, and fluctuating in patients with cCMV [28]. The unpredictable nature of these serious sequelae is likely to contribute to or exacerbate the stress experienced by caregivers. Other pediatric diseases, such as type I diabetes, have also been shown to be associated with stress, anxiety, and depression in patients and their caregivers [29]. Parents of children with cCMV have reported feelings of guilt about the child’s infection, stress associated with the responsibility of managing the child’s condition, worries about their child’s future, impacts on relationships with their other children and/or partners, difficulties in social relationships, and impacts on work and family finances [18]. A cross-sectional study in the United Kingdom (UK) used questionnaires to measure HRQoL in 70 families with children with cCMV, and reported that cCMV had significant effects on HRQoL of children with cCMV and their parents [30]. This is broadly consistent with the findings presented here, although the UK study focused on quantitative assessment of HRQoL, whereas the present study focused on understanding concepts describing the lived experiences of caregivers and caregivers’ perceptions of patients’ experiences. A study in the UK reported that lack of knowledge about cCMV among medical professionals could contribute to parents’ distress, and uncertainty about long-term outcomes added to the emotional burden [31]. A survey of pediatric care providers in the US found that respondents felt they had low knowledge of cCMV [32], consistent with the perceptions of some caregivers in the present study. This lack of awareness highlights the importance of targeted education and information programs, including healthcare professionals, pregnant women and the general population.

Participants in this study described few financial burdens from cCMV, but this may not be generalizable to households with different financial circumstances. A static cost model developed for the UK estimated the cost of cCMV in 2016 at £732 million, of which 60% related to indirect costs such as lost productivity and costs probably borne by families, such as specialized accommodation and respite care for carers [33].

Results reported in the present study support and build on findings from earlier qualitative work, including a previous conceptual model published in abstract form [22]. The HRQoL dimensions captured in this previous model (e.g., emotional, physical, social, work) are similar to those identified in the current model and are organized similarly, distinguishing between long-term symptoms and transient ones at birth. The earlier model has some limitations, as it was based on a literature review and patient/caregiver-reported information gathered through social media posts [22]. Our study builds on the findings of this earlier work by collecting primary data and descriptions of their experiences from caregivers of patients with cCMV. Facilitators and barriers to caregivers’ ability to cope identified in a small qualitative study in the UK mirror some of the modifying factors in the current model (e.g., social network) [31].

The final conceptual model developed from interview findings in this study could help healthcare professionals to better understand the lived experience of children with cCMV and their caregivers and the challenges they face [34]. Medical professionals’ knowledge and awareness of cCMV was mentioned as a positive experience by nine caregivers (36%) in the present study and a negative experience by eight (32%). Improving medical professionals’ understanding of the disease experience could potentially help to make this a positive factor for more families affected by cCMV, which could be a valuable first step in improving their lives.

The final conceptual model could inform development of a disease-specific clinical outcome assessment tool to describe and quantify cCMV burden of disease in patients and caregivers [34]. However, the wide range of experiences uncovered in this study may present a challenge in developing a tool that can accommodate the variety of impacts encountered by different families.

A key strength of this study was the overall ethnic diversity of the sample and its mix of male and female caregivers. It was also geographically diverse within the US and included participants with a range of education and employment status. Saturation analysis indicated that the sample size of 25 was sufficient for the study objectives and is consistent with sample sizes reported to achieve concept saturation in qualitative research [35]. The study also has some limitations. It was conducted in the US and may not be generalizable to other geographic regions. The study may not have captured all caregiver-reported patient impacts or caregiver impacts, and, as patients were not interviewed, it did not directly reflect the patient perspective. Overall, study participants described few financial problems from cCMV, and their level of financial stability may not be generalizable to households with different insurance status or financial circumstances. No interviews were conducted with caregivers of adult patients with cCMV, with asymptomatic patients who did not develop sequelae or long-term complications, or with caregivers of patients with severe long-term complications (e.g., severe intellectual disability or death); and therefore, the study may not have captured the impact of cCMV in these groups. The study was also limited to patients who survived the initial phase of cCMV and therefore it could not capture the impact of deaths due to cCMV. The sample was selected from parents who had lived with the child with cCMV since birth, and therefore may not be representative of the disease burden experienced by other types of caregivers. Further research would be valuable to capture the experience of adult patients with cCMV, and to interview patients with cCMV directly where possible and ethical to do so. Larger studies collecting self-reported data may help to explore the generalizability of the results of this study.

## Conclusions

This study advances current understanding of caregiver-reported patient impacts and caregiver experiences related to cCMV, including the burden of disease and impacts on HRQoL. These results and the final conceptual model could be used to support the development of a disease-specific clinical outcome assessment tool to further describe and quantify the burden of cCMV disease in future studies.

## Supplementary Information

Below is the link to the electronic supplementary material.

Supplementary Material 1

## Abbreviations

cCMV: Congenital cytomegalovirus
CMV: Cytomegalovirus
HIPAA: Health Insurance Portability and Accountability Act
HRQoL: Health-related quality of life
SLR: Systematic literature review
TLR: Targeted literature review
UK: United Kingdom
US: United States

## Declarations

All data used for this publication was received by GSK. For access to anonymized subject level data, please contact GSK. Additional information on the study materials can be provided on request.
