# Supplementary material for: Understanding the burden of congenital cytomegalovirus (cCMV) infection: concept elicitation interviews with caregivers of pediatric cCMV patients and development of a conceptual disease model
Source: Qual Life Res. 2026 Mar 1;35(4):79. doi: 10.1007/s11136-025-04142-3 (PMC12950654; doi:10.1007/s11136-025-04142-3)
Supplement: Supplementary file 1 — Supplementary Material 1 [file 11136_2025_4142_MOESM1_ESM.docx]

Supplementary Information Title page

**Understanding the burden of congenital cytomegalovirus (cCMV) infection: Concept elicitation interviews with caregivers of pediatric cCMV patients and development of a conceptual disease model**

Brooke M. Currie^1^, Georges Van Kriekinge^2^, Aurélie Pimienta^2,5^, Cory D. Saucier^3^, KD Jacobs^3^, Laura Tesler Waldman^3, 6^, Christine M. Salvatore^4^

^1^ GSK, Collegeville, PA, USA; brooke.m.currie@gsk.com

^2^ GSK, Wavre, Belgium; GVK georges.m.van-kriekinge@gsk.com

^3^ QualityMetric, Johnston, RI, USA; CDS csaucier@qualitymetric.com, KDJ kjacobs@qualitymetric.com

^4^ Weill Cornell Medicine, New York, NY, USA; chs2032@med.cornell.edu

^5^ Current affiliation: RTI Health Solutions, Ann Arbor, MI, USA; apimienta@rti.org

^6^ Current affiliation: Lumanity Patient-Centered Outcomes LLC, Boston, MA, USA, laura.waldman@lumanity.com

**Corresponding author:**

Brooke M. Currie

1250 S Collegeville Rd, Collegeville, PA 19423, USA

Tel: +1 202-306-0630

Email: brooke.m.currie@gsk.com

## Supplementary Information S1. Supplementary methods, and information on authors

No-one was present other than the participant and interviewer, and there were no repeat interviews. Transcripts were not returned to the participants and participants did not provide feedback on the findings. There was no relationship between researchers and participants before the study. The informed consent form and recruitment materials contained information about the study goals and the reasons for the research. The final data source for analysis included all interview transcripts and interviewer-recorded field notes.

**Information on authors**

|  | **BMC** | **GVK** | **AP** | **CDS** | **KDJ** | **LTW** | **CMS** |
| --- | --- | --- | --- | --- | --- | --- | --- |
| **Credentials** | MPH | N/A | MS, MA | MPH | PhD | PhD | MD |
| **Occupation at time of study** | GSK employee | GSK employee | GSK employee | QM employee | QM employee | QM employee | Weill Cornell Medicine, Pediatric Infectious Diseases |
| **Gender** | Female | Male | Female | Male | Female | Female | Female |
| **Experience and training** | Graduate degree in Public Health and 15+ years of experience conducting and reviewing qualitative research | GSK researcher | BSc, MSc in psychology, Licensed psychologist, MSc in Health Economics | Degrees in Psychology and Public Health | Degrees in Public Health | Degrees in cultural and medical anthropology | MD, Pediatric infectious diseases specialist |

BSc, bachelor of science; MD, doctor of medicine; MPH, master of public health; MSc, master in science; PhD, doctor of philosophy; QM, QualityMetric

## Supplementary Figure S1 Preliminary cCMV conceptual model informed by targeted literature review

**
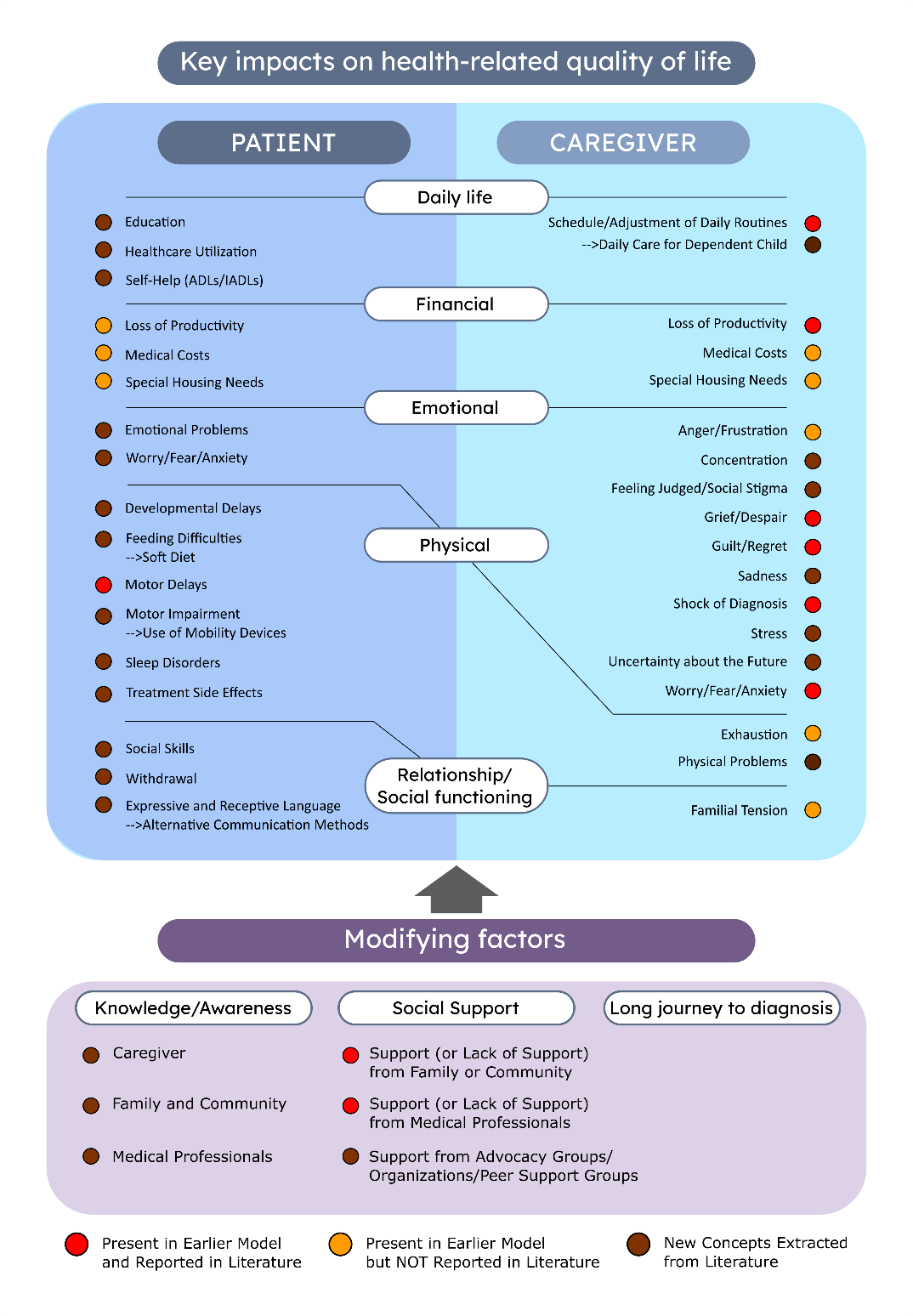
**

ADL, activities of daily living; cCMV, congenital cytomegalovirus; IADL, instrumental activities of daily living

## Supplementary Figure S2 PRISMA diagram for targeted literature review


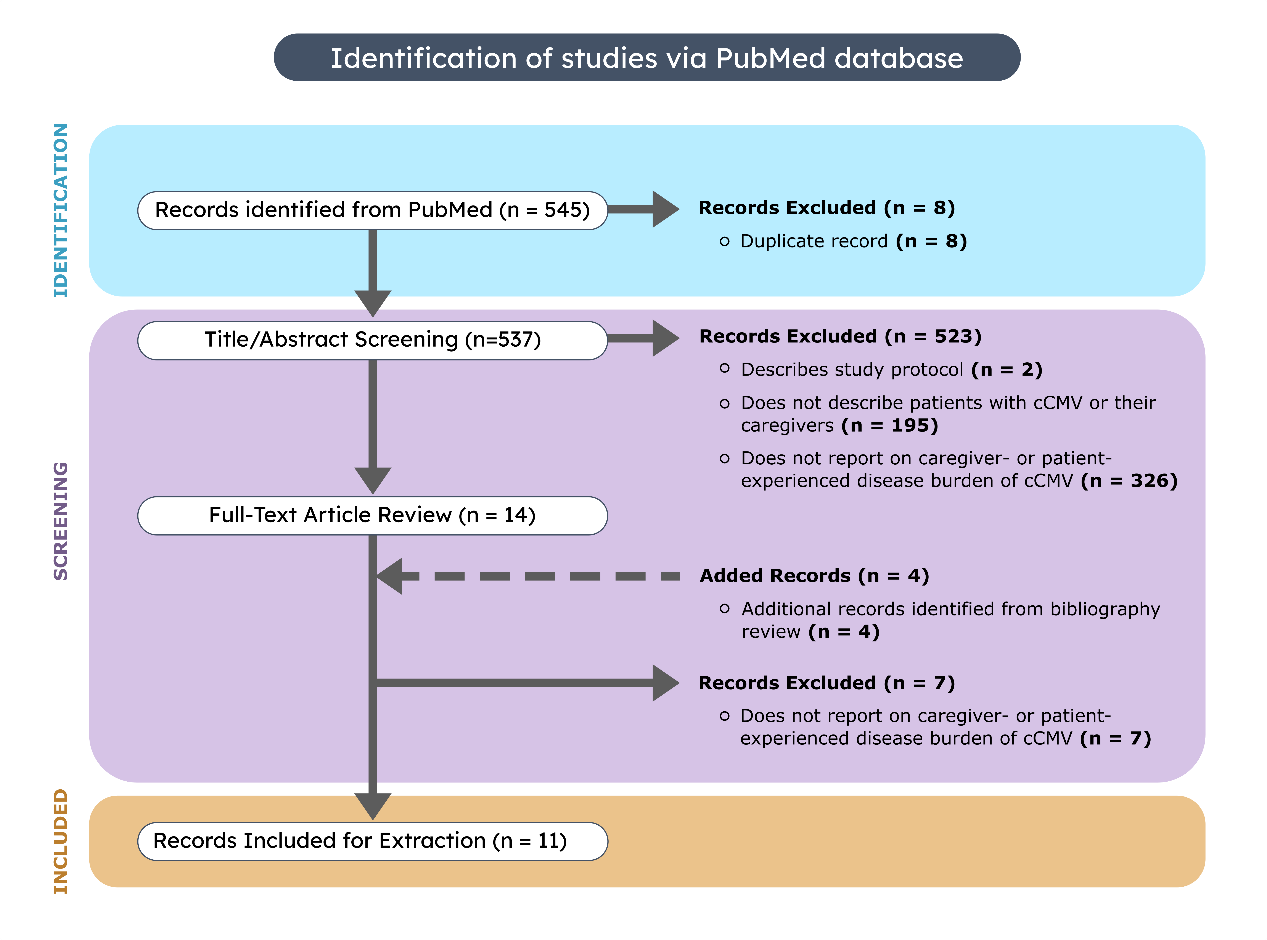


cCMV, congenital cytomegalovirus; PRISMA; Preferred Reporting Items for Systematic reviews and Meta-Analyses

## Supplementary Table S1. PubMed search strategy and inclusion criteria for targeted literature review

| **PubMed search string** |
| --- |
| (“congenital cytomegalovirus”[TiAb] OR “cCMV”[TiAb] OR “cCMVi”[TiAb] OR ("Cytomegalovirus Infections/congenital"[MeSH]) OR ("Cytomegalovirus"[MeSH] AND “congenital”[TiAb])) |
| **AND** |
| (“quality of life”[TiAB] OR “QoL”[TiAb] OR “health-related quality of life”[TiAb] OR “health related quality of life”[TiAb] OR “HRQoL”[TiAb] OR “HRQL”[TiAb] OR “burden*”[TiAb] OR “impact*”[TiAb] OR “function*”[TiAb] OR “daily activit*”[TiAb] OR “well-being”[TiAb] OR “emotional*”[TiAb] OR “impairment*”[TiAb] OR “productivity”[TiAb] OR “financ*”[TiAb] OR “economic*”[TiAb] OR “social*”[TiAb] OR “relationship*”[TiAb] OR “caregiver*”[TiAb] OR “qualitative”[TiAb] OR “focus group*”[TiAb] OR “interview*”[TiAb] OR “survey*”[TiAb] OR “mother*”[TiAb] OR “parent*”[TiAb]) |
| **AND** |
| English[Language] |
| **AND** |
| (“2012”[Date – Publication]: “2022”[Date - Publication]) |
| **AND** |
| “Humans”[Species] |
| **Inclusion criteria** |
| The article describes a study for which the sample includes (or describes) patients with cCMV of all age groups (e.g., infant, children, adults), or their caregivers  The article is available in English  The article is published in a peer-reviewed journal in 2012 or later  The article does not describe a study protocol  The article is not a duplicate record  The article reports on caregiver- or patient-experienced disease burden of cCMV (e.g., impacts on quality of life) |

cCMV, congenital cytomegalovirus; cCMVi, congenital cytomegalovirus infection; HRQL, health-related quality of life; HRQoL, health-related quality of life; MeSH, Medical Subject Headings; QoL, quality of life

## Supplementary Table S2. Patient- and caregiver-experienced impacts of cCMV on HRQoL, and modifying factors, identified in the targeted literature review

| **Domain** | **Key impact** | **Count** |
| --- | --- | --- |
| **Patient-experienced impacts** | | |
| Physical | Treatment Side Effects | 1 |
|  | Use of Mobility Devices | 1 |
|  | Feeding Difficulties* | 2 |
|  | Motor Delays | 3 |
|  | Developmental Delays* | 2 |
|  | Soft Diet* | 1 |
|  | Motor Impairment | 1 |
|  | Sleep disorders | 1 |
|  | **Total physical** | **12** |
| Social | Alternative Communication Strategies | 2 |
|  | Social Skills* | 1 |
|  | Expressive and Receptive Language | 6 |
|  | Withdrawal | 1 |
|  | **Total social** | **10** |
| Role | Education | 3 |
|  | Self-Help* (Autonomy) | 1 |
|  | **Total role** | **4** |
| Emotional | Emotional Problems* | 1 |
|  | Worry/Fear/Anxiety | 1 |
|  | **Total emotional** | **2** |
| Additional impacts | Healthcare utilization | 3 |
| **Total (all domains)** |  | **31** |
| **Caregiver-experienced impacts** | | |
| Emotional | Guilt/Regret | 3 |
|  | Worry/Fear/Anxiety | 3 |
|  | Grief/Despair | 2 |
|  | Uncertainty about the Future | 2 |
|  | Stress | 1 |
|  | Sadness | 1 |
|  | Feeling Judged/Social Stigma | 1 |
|  | **Total emotional** | **13** |
| Role | Daily Care for Dependent Child | 2 |
|  | Schedule/Adjustment of Daily Routine | 1 |
|  | **Total role** | **3** |
| Financial | Loss of productivity | 1 |
| Physical | Physical problems* | 1 |
| Additional impacts | Concentration* | 1 |
|  | Shock of Diagnosis | 1 |
|  | **Total additional impacts** | **2** |
| **Total (all domains)** |  | **20** |
| **Modifying factors** | |  |
| Long Journey to Diagnosis | | 2 |
| Support (or Lack of Support) from Professionals | | 2 |
| Support (or Lack of Support) from Family and Community | | 2 |
| Support from Advocacy Groups/Organizations/Peer Support Groups | | 2 |
| Awareness in Medical Community | | 1 |
| Parental Knowledge | | 1 |
| Family and Community Knowledge or Awareness | | 1 |
| **Total** | | **11** |

cCMV, congenital cytomegalovirus, HRQoL, health-related quality of life

* Broad or generalized term used within the literature without sufficient context or detailed interpretation

## Supplementary Table S3. Modifying factors reported by caregivers of children with cCMV

| **Modifying factor** | **Number (n=25)** | **%** |
| --- | --- | --- |
| Awareness/Knowledge | 25 | 100 |
| Social Support | 24 | 96.0 |
| Coping | 19 | 76.0 |
| Time Since Diagnosis | 18 | 72.0 |
| Insurance/Financial Stability | 14 | 56.0 |
| Journey to Diagnosis | 12 | 48.0 |
| Treatment/Therapy | 12 | 48.0 |

cCMV, congenital cytomegalovirus
